# Editorial: Psychiatric diagnoses: current state and methodological issues

**DOI:** 10.3389/fpsyt.2023.1194755

**Published:** 2023-04-13

**Authors:** Julie Nordgaard, Luis Madeira, Ann K. Shinn, Michel Cermolacce

**Affiliations:** ^1^Mental Health Center Amager, Copenhagen, Denmark; ^2^Department of Clinical Medicine, University of Copenhagen, Copenhagen, Denmark; ^3^Department of Psychiatry, Institute of Preventive Medicine, University of Lisbon, Lisbon, Portugal; ^4^Psychotic Disorders Division, McLean Hospital, Belmont, MA, United States; ^5^Department of Psychiatry, Harvard Medical School, Boston, MA, United States; ^6^Department of Psychiatry, APHM and Aix Marseille University, Marseilles, France

**Keywords:** nosology, reliability, assessment, interview, phenomenology

Lack of consensus about diagnoses continues to haunt psychiatry, and efforts to improve the reliability and validity of diagnoses have unfortunately not led to major advances. The operational criteria introduced with the DSM-III in 1980 and adopted in ICD-10 in 1992 did not fulfill the promise of significant advances in etiological and therapeutic knowledge of mental disorders. In this deadlock, alternative approaches have been suggested. For example, dimensional approaches to classifying mental disorders, e.g., Research Domain Criteria (RDoC) or Hierarchical Taxonomy of Psychopathology (HiToP), have been put forth as an alternative to the conventional categorical diagnoses. Another line of research, by contrast, points to the importance of diagnostic categories and suggests an approach based on diagnostic prototypes. These nosological questions are intrinsically bound to a range of fundamental issues in psychiatry that contribute to the current differential diagnostic confusions—e.g., questions about the appropriate methodology for assessing psychopathology, a lack of conceptual clarity of many psychopathological phenomena, and a general decline in knowledge of psychopathology during the last decades.

In this collection, Lindhardt et al., Neto et al., and Wood et al. examined the stability and reliability of diagnostic assessments. In a register study, Lindhardt et al. followed the course of young people who sought help from an early psychosis detection service but were assessed by this service not to have psychosis (i.e., the non-cases). During follow-up, 15% of the non-cases were diagnosed with psychosis. Moreover, both cases and non-cases had markedly impaired social functioning. The findings indicate a significant unmet mental health service need in a large subset of young help-seeking people. Additionally, it shines a light on the challenges relating to ascertainment of cases and non-cases, raising questions about what constitutes a sufficient assessment and who should conduct such assessments.

Neto et al. compared diagnoses allocated by the same psychiatric residents: first, a clinical diagnosis without a sharp focus on diagnostic criteria, and, second, a research-diagnosis made by rigorously adhering to ICD-10 criteria. They included patients with a range of different clinical diagnoses. The results demonstrated a moderate agreement between the single raters' clinical diagnoses and the research diagnoses. This points to medical doctors adhere closer to the ICD-10 criteria in their clinical work than the researchers had suspected.

While Lindhardt et al. and Neto et al. examined diagnostic ascertainment in clinical settings, Wood et al. investigated diagnostic stability in a research sample. They found that among 142 individuals with psychosis who underwent diagnostic assessments with a Structured Clinical Interview for DSM (SCID) on at least two occasions, diagnostic change occurred in 25%. There were variable rates of diagnostic stability across psychotic disorders diagnoses, with bipolar I disorder showing the highest and psychotic disorder not otherwise specified and schizophreniform disorder showing the lowest stability. These findings suggest that diagnostic interviews, even when conducted in a highly operationalized and systematic way in a research setting, may misclassify a sizable minority of study subjects.

Larsen et al. deliver a more sweeping appraisal of the contemporary approach to psychiatric diagnosis, critiquing in particular the ways in which both existing conceptual models and care delivery system approach dual diagnosis, or comorbid psychiatric and addictive disorders. They offer Enactive Psychiatry, which draws on several theoretical approaches (embodiment, system theory, and ecological psychology), as a more integrative lens through which psychiatric diagnoses can be better conceptualized. They propose the importance of considering the complex and dynamic interplay between the brain and body as well as how the whole person is embedded in their extended environment and socially medicated drug use influence the individual patient.

Looking more closely at psychopathological phenomena, Rasmussen et al. examined anomalies of imagination and self-disorders among people with schizophrenia spectrum disorders and compared them with controls. All were examined using semi-structured and phenomenologically oriented instruments, showing high inter-rater reliability despite the subjective nature of reported experiences. Both anomalies of imagination and self-disorders aggregated significantly within the schizophrenia spectrum and the authors consider them overlooked, but characteristic phenomena of the schizophrenia spectrum.

Hu et al. examined the phenomenon of Hikikomori, which has only recently been described. Hikikomori is a condition of severe social withdrawal which was first described in Japan but has since been reported in several other countries. Hu et al. developed a standardized self-questionnaire to screen for Hikikomori. Students and teachers at Chinese universities and colleges were invited to participate in screening for Hikikomori in the nationwide survey. The screening showed a relatively high prevalence of Hikikomori, indicating the importance of closer examination of the phenomenon as well as delineation from other mental disorders such as schizophrenia spectrum disorders and depression.

Contributing to a way forward, Messas et al. offer a coherent description on how to establish a novel interview methodology, aiming at improving both validity and reliability of the psychiatric diagnoses. This includes harmonizing the first and second person perspectives through a hermeneutical stage in which narratives are transformed into a specific scientific object. By raising awareness of dialectics and phenomenological psychopathology, they consider it possible to scientifically explore pre-reflexive alterations of lived experience with relevance for diagnosis.

Gozé also draws on insights from phenomenology and aims to re-conceptualize and re-actualize the classic concept of Praecox Gefühl. Gozé argues that this concept is more complementary than contradictory with an operationalized diagnostic approach, by coupling implicit and explicit levels of medical judgement. This has implications for handling this feeling when encountered in the clinical meeting with the patient, and it may also be of crucial importance in terms of clinical education.

Collectively, the articles included in this Research Topic cast light on the challenges of psychiatric diagnoses while also offering potential ways forward. We hope that the Research Topic dedicated to psychiatric diagnosis has contributed to a much-needed discussion and will further stimulate new ideas and research to advances in psychiatric nosology.

## Author contributions

JN wrote the first draft. All authors contributed to revise the manuscript and approved the submitted version.

